# Supplementary material for: Genetic hypogonadal mouse model reveals niche-specific influence of reproductive axis and sex on intestinal microbial communities
Source: Biol Sex Differ. 2023 Nov 6;14:79. doi: 10.1186/s13293-023-00564-1 (PMC10626657; doi:10.1186/s13293-023-00564-1)
Supplement: Supplementary file 1 — Additional file 1: Table S1. Location, sex and hpg genotype affect compositional differences between samples. P values, degrees of freedom (DF), sum of squares (SumOFSqs), R2, and F statistics from mixed-effect model PERMANOVA for effects of section, sample type (lumen vs. mucosa), sex, and hpg genotype. P values < 0.05 shown in bold. Table S2. R2 and p values from mixed-effect PERMANOVAs for unweighted UniFrac distances. P values < 0.05 shown in bold. Table S3. Effect of section, sample type, sex, or hpg genotype on top 10 most abundant bacterial families clr-transformed abundances. FDR-corrected p values shown for linear mixed-effect model. Fixed effects were section*sample type*sex*hpg. Non-significant interactions were removed. P values < 0.05 shown in bold. Figure S1. Rarefaction curves by intestinal section. A Duodenum, B Ileum, and C Cecum. Figure S2. Source contribution to feces by hpg genotype and sex. 95% confidence intervals shown for SourceTracker source proportions of feces (sink) samples by sex and hpg genotype. Figure S3. Beta diversity differences between lumen and mucosa. A NMDS orientation plots of Euclidean distances comparing lumen and mucosa in the duodenum, ileum, and cecum. B NMDS plots of unweighted UniFrac distances for the duodenum, ileum, and cecum. The clr-transformed counts of genera were fit to each ordination and arrows are the vector average of the genus. Genera shown had the top 10 R2 values of significant genera fit to the ordination (FDR-corrected p values < 0.05 determined by permutation test). Figure S4. NMDS ordination of Euclidean distances based on bacterial family abundances for each intestinal environment. Figure S5. Family relative abundance for each individual sample. Relative abundance of each family for A duodenum, B ileum, and C cecum. Each bar represents a mouse intestinal sample. Figure S6. Strength and complexity of genus-level balance models. Sex differences in wild-type and mutant mice and hpg genotype differences [file 13293_2023_564_MOESM1_ESM.docx]

**Genetic hypogonadal mouse model reveals niche-specific influence of reproductive axis and sex on intestinal microbial communities**

This additional data file contains:

Additional Tables 1-3

Additional Figures 1-7

| Fixed Effect | p value |  | Df | SumOfSqs | R2 | F |
| --- | --- | --- | --- | --- | --- | --- |
| Section | **0.0001** |  | 2 | 464289 | 0.1953 | 70.5253 |
| Sample Type | **0.0001** |  | 2 | 110964 | 0.0467 | 16.8554 |
| Sex | **0.0134** |  | 1 | 8017 | 0.0034 | 2.4355 |
| Genotype | **0.0004** |  | 1 | 14102 | 0.0060 | 4.2840 |
| Sex:Genotype | **0.0065** |  | 1 | 9119 | 0.0038 | 2.7702 |
| Sex:Sample Type | 0.5466 |  | 2 | 5900 | 0.0025 | 0.8962 |
| Genotype:Sample Type | 0.8430 |  | 2 | 4946 | 0.0021 | 0.7514 |
| Sex:Section | 0.2070 |  | 2 | 7614 | 0.0032 | 1.1566 |
| Genotype:Section | **0.0473** |  | 2 | 10197 | 0.0043 | 1.5489 |
| Sample Type:Section | **0.0002** |  | 2 | 27763 | 0.0117 | 4.2172 |
| Sex:Genotype:Sample Type | 0.2931 |  | 2 | 7019 | 0.0030 | 1.0662 |
| Sex:Genotype:Section | 0.2066 |  | 2 | 7542 | 0.0032 | 1.1457 |
| Sex:Sample Type:Section | 0.6726 |  | 2 | 5424 | 0.0023 | 0.8240 |
| Genotype:Sample Type:Section | 0.8259 |  | 2 | 4993 | 0.0021 | 0.7585 |
| Sex:Genotype:Sample Type:Section | 0.2861 |  | 2 | 7064 | 0.0030 | 1.0730 |
| Residual |  |  | 511 | 1682031 | 0.7076 |  |
| Total |  |  | 538 | 2376984 | 1 |  |

**Table S1 | Location, sex and *hpg* genotype affect compositional differences between samples.** P values, degrees of freedom (DF), sum of squares (SumOFSqs), R2, and F statistics from mixed-effect model PERMANOVA for effects of section, sample type (lumen vs. mucosa), sex, and *hpg* genotype. P values < 0.05 shown in bold.

| Fixed Effect | Duodenum | | Ileum | | Cecum | |
| --- | --- | --- | --- | --- | --- | --- |
|  | P value | R2 | P value | R2 | P value | R2 |
| Sample Type | **0.0001** | 0.0811 | **0.0001** | 0.0512 | **0.0014** | 0.0173 |
| Sex | 0.1687 | 0.0079 | **0.0198** | 0.0150 | 0.0745 | 0.0103 |
| Genotype | **0.0016** | 0.0175 | **0.0257** | 0.0139 | **0.0182** | 0.0122 |
| Sex:Genotype | 0.1587 | 0.0080 | 0.1146 | 0.0093 | **0.0105** | 0.0134 |
| Sex:Sample Type | 0.0723 | 0.0095 | 0.9800 | 0.0023 | 0.9097 | 0.0033 |
| Genotype:Sample Type | 0.1964 | 0.0075 | 0.0864 | 0.0102 | 0.9172 | 0.0033 |
| Sex:Genotype:Sample Type | 0.1752 | 0.0078 | 0.1977 | 0.0079 | 0.9905 | 0.0019 |

**Table S2. R2 and p values from mixed-effect PERMANOVAs for unweighted UniFrac distances**. P values < 0.05 shown in bold.

| Fixed Effect | Bacteroidaceae | Clostridiaceae | Deferribacteraceae | Eggerthellaceae | Lachnospiraceae |
| --- | --- | --- | --- | --- | --- |
| Sample Type | **1.158E-04** | 1.053E-01 | **1.067E-06** | **1.447E-25** | **2.709E-03** |
| Section | **1.068E-79** | **0.000E+00** | **3.456E-50** | **1.279E-22** | **6.520E-36** |
| Genotype | 0.7974 | 0.5403 | 0.3247 | **0.0033** | 0.7974 |
| Sex*Genotype | 0.7119 | 0.7475 | 0.9501 | 0.7119 | 0.7119 |
| Genotype*Section | **0.0056** | 0.5062 | 0.5062 | 0.3226 | 0.6400 |
| Sample Type*Section | **1.267E-03** | **1.267E-03** | 1.271E-01 | **2.005E-08** | **7.914E-04** |
| Sex*Genotype*Sample Type*Section | 0.4384 | 0.2403 | 0.2403 | 0.0712 | 0.0715 |
|  |  |  |  |  |  |
| Fixed Effect | **Lactobacillaceae** | **Muribaculaceae** | **Oscillospiraceae** | **Rikenellaceae** | **Ruminococcaceae** |
| Sample Type | **2.504E-53** | **8.330E-30** | 1.870E-01 | **1.695E-03** | 0.1382 |
| Section | **2.472E-173** | **5.422E-22** | **6.587E-39** | **9.833E-114** | **4.912E-27** |
| Genotype | 0.1031 | **1.627E-05** | 0.9377 | 0.5142 | 0.5142 |
| Sex*Genotype | 0.8381 | **0.0401** | 0.7119 | 0.7119 | 0.7119 |
| Genotype*Section | 0.6400 | **0.0056** | 0.9392 | **0.0056** | 0.6400 |
| Sample Type* Section | **2.602E-19** | **8.596E-15** | **4.180E-02** | **1.480E-02** | **1.942E-03** |
| Sex*Genotype*Sample Type*Section | 0.0712 | **0.0124** | 0.1690 | 0.2403 | 0.2403 |

**Table S3 | Effect of section, sample type, sex, or *hpg* genotype on top 10 most abundant bacterial families clr-transformed abundances.** FDR-corrected p values shown for linear mixed-effect model. Fixed effects were section*sample type*sex**hpg*. Non-significant interactions were removed. P values < 0.05 shown in bold.


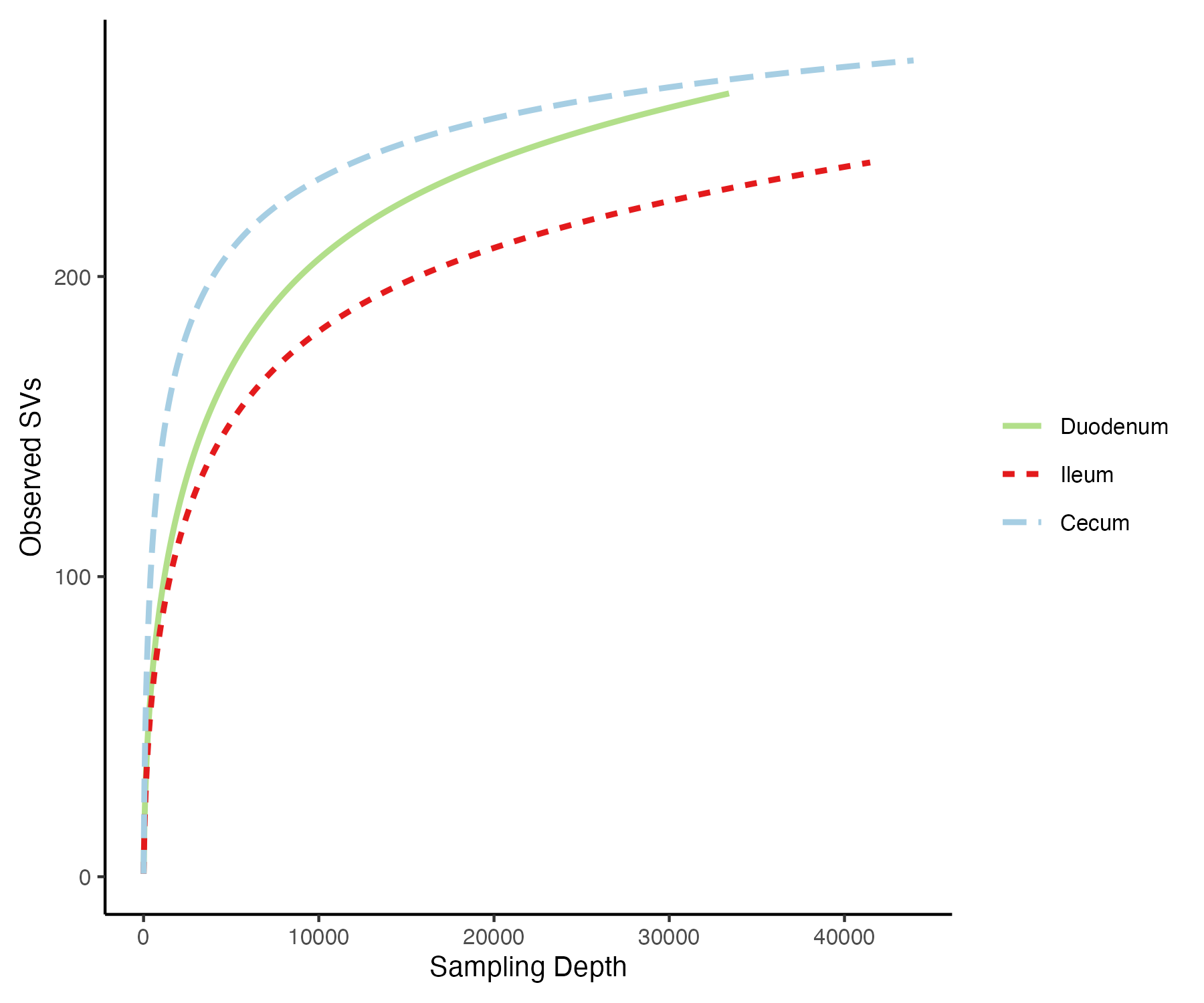


**Figure S1 |** **Rarefaction curves by intestinal section. A** Duodenum, **B** Ileum, and **C** Cecum.


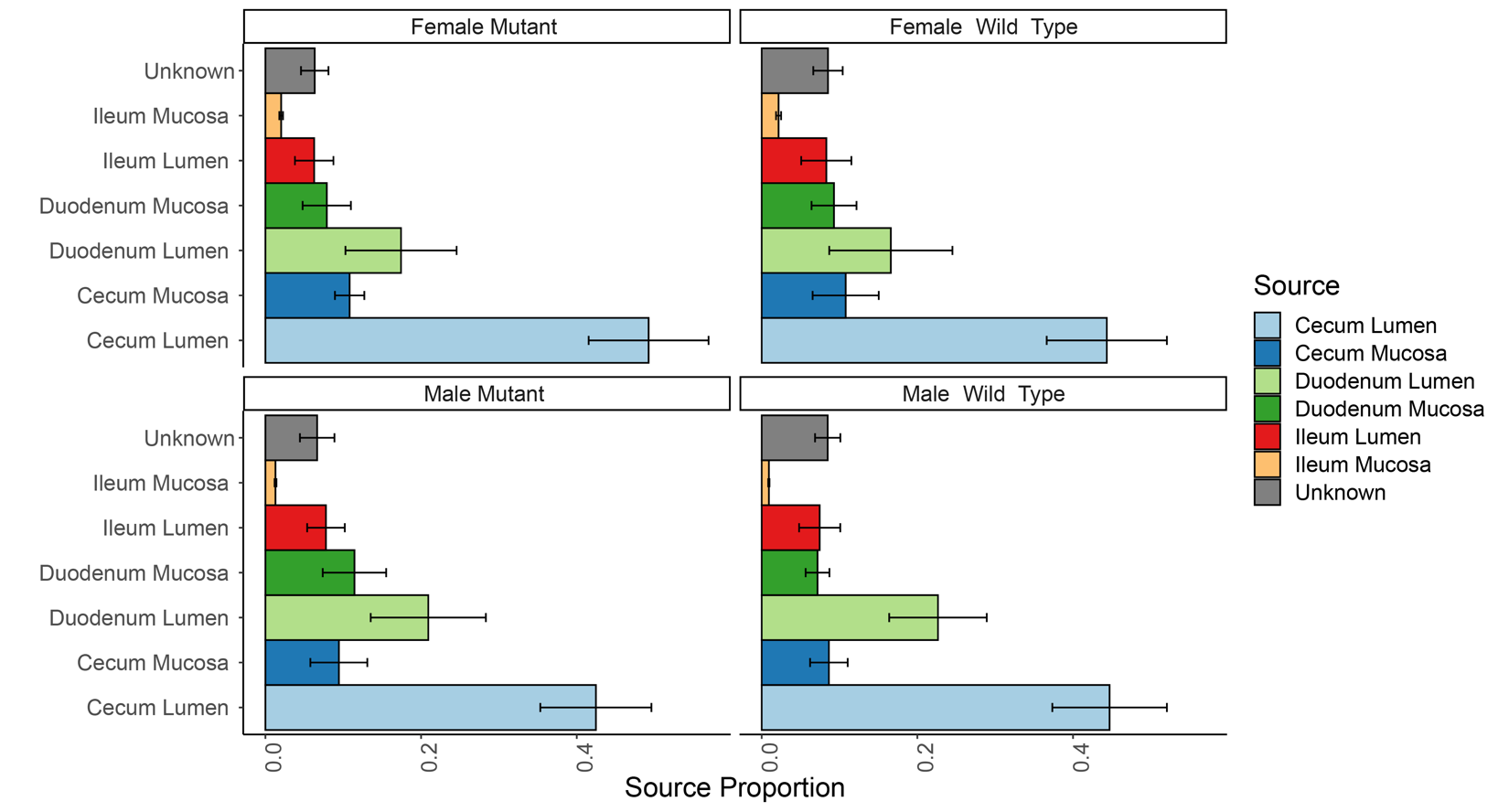


**Figure S2 |Source contribution to feces by *hpg* genotype and sex**. 95% confidence intervals shown for SourceTracker source proportions of feces (sink) samples by sex and *hpg* genotype.

**
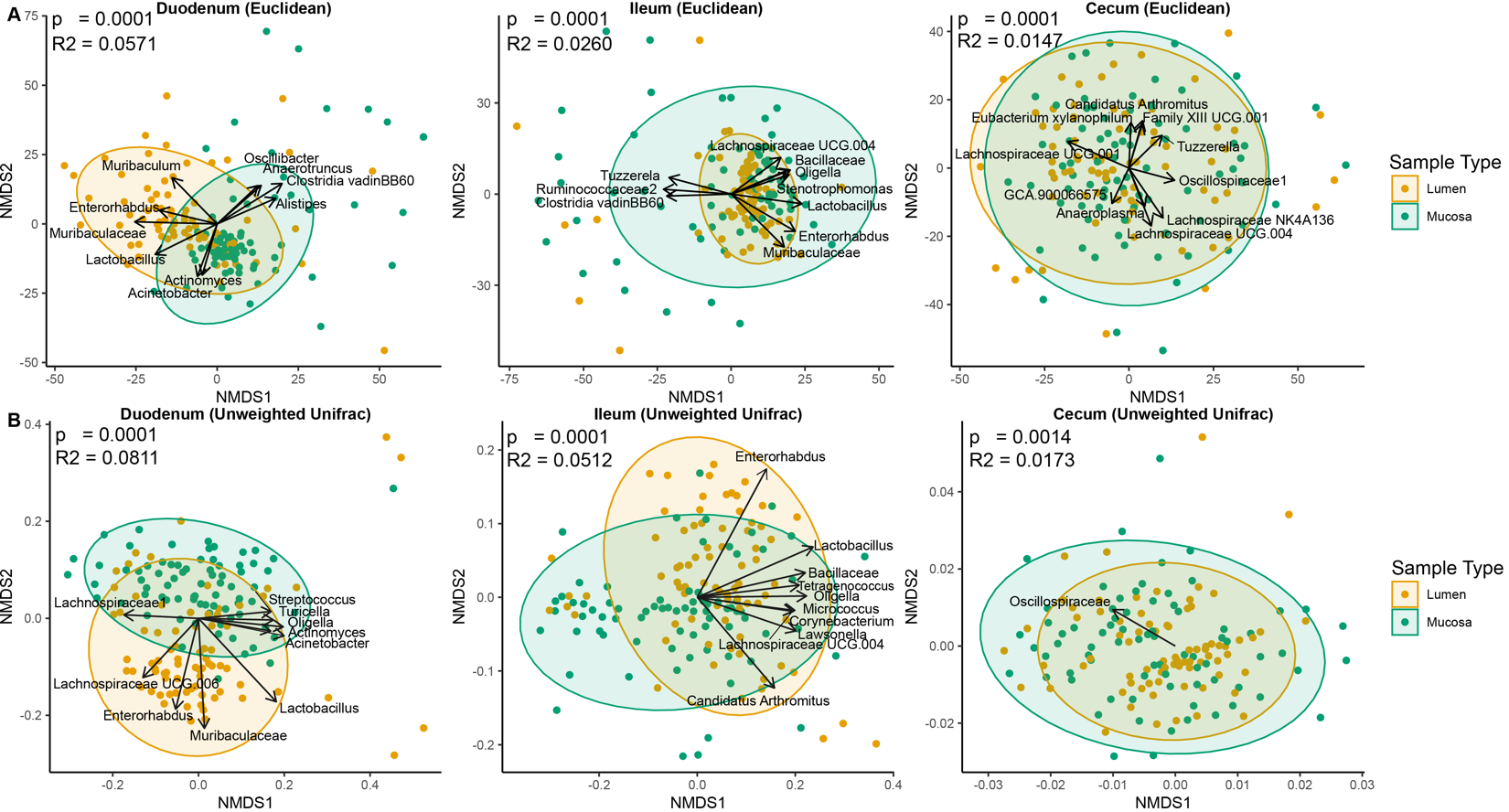
**

**Figure S3 |** **Beta diversity differences between lumen and mucosa**. **A** NMDS orientation plots of Euclidean distances comparing lumen and mucosa in the duodenum, ileum, and cecum. **B** NMDS plots of unweighted UniFrac distances for the duodenum, ileum, and cecum. The clr-transformed counts of genera were fit to each ordination and arrows are the vector average of the genus. Genera shown had the top 10 R^2^ values of significant genera fit to the ordination (FDR-corrected p values < 0.05 determined by permutation test).


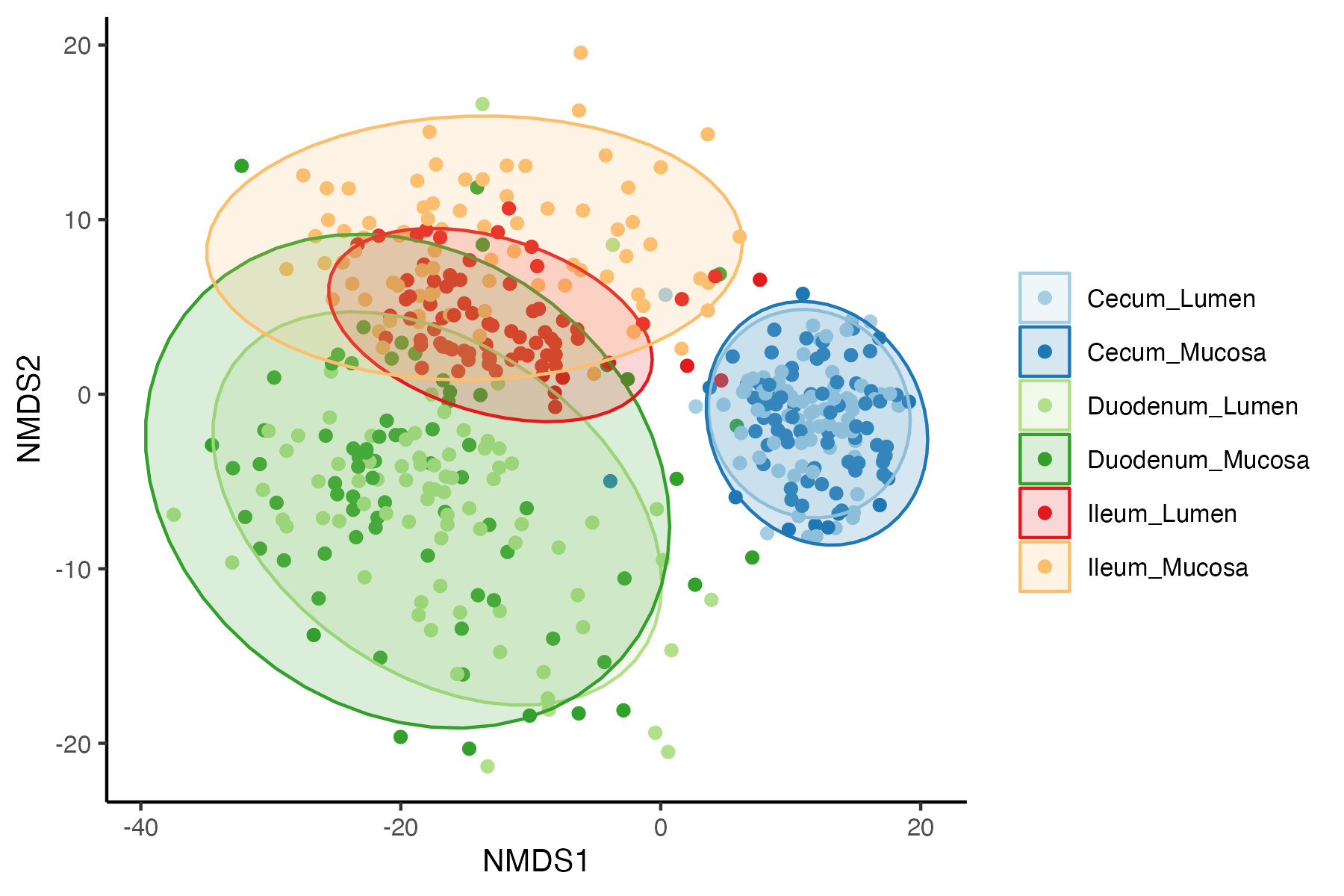


**Figure S4 |** NMDS ordination of Euclidean distances based on bacterial family abundances for each intestinal environment.

**
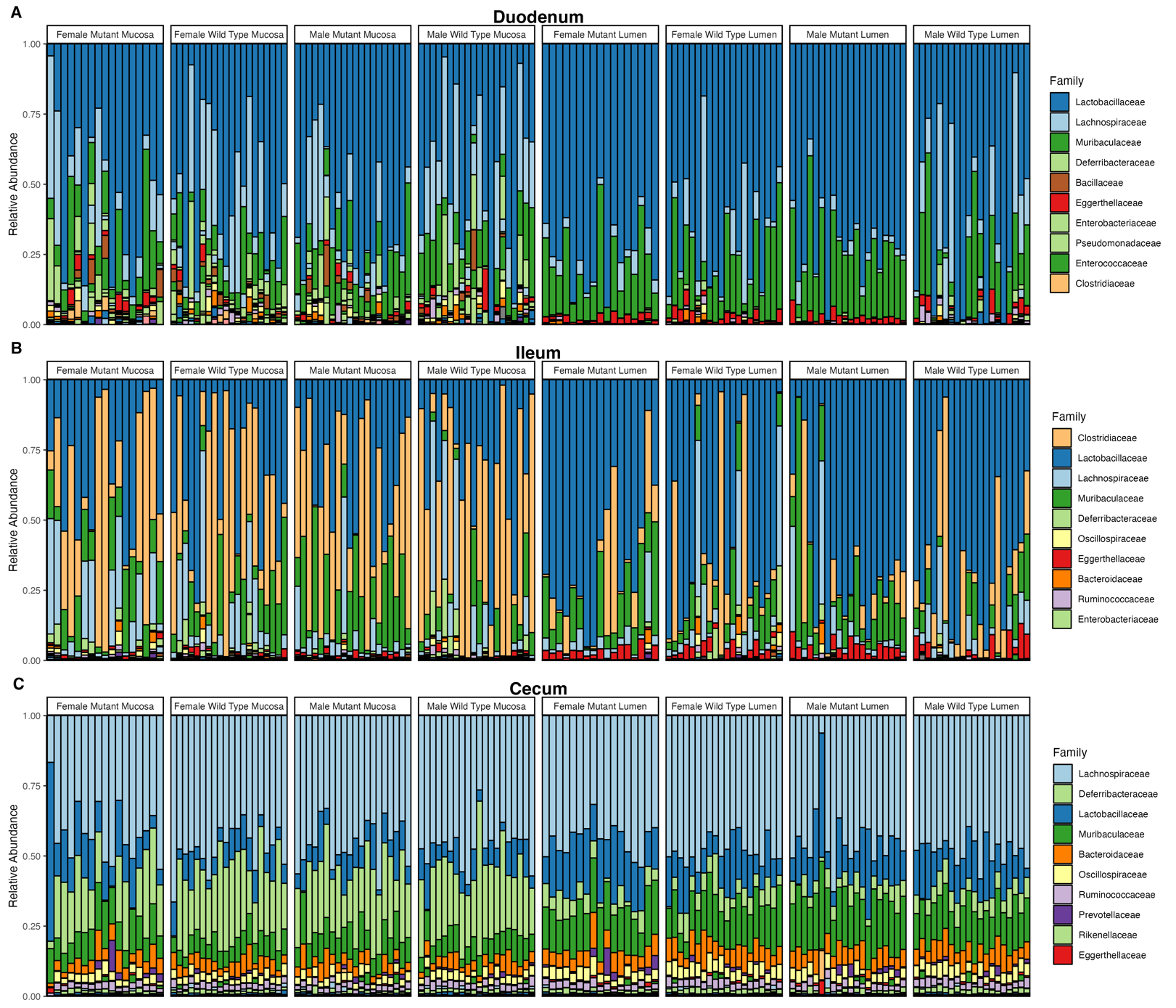
**

**Figure S5 |Family relative abundance for each individual sample**. Relative abundance of each family for **A** duodenum, **B** ileum, and **C** cecum. Each bar represents a mouse intestinal sample.

**
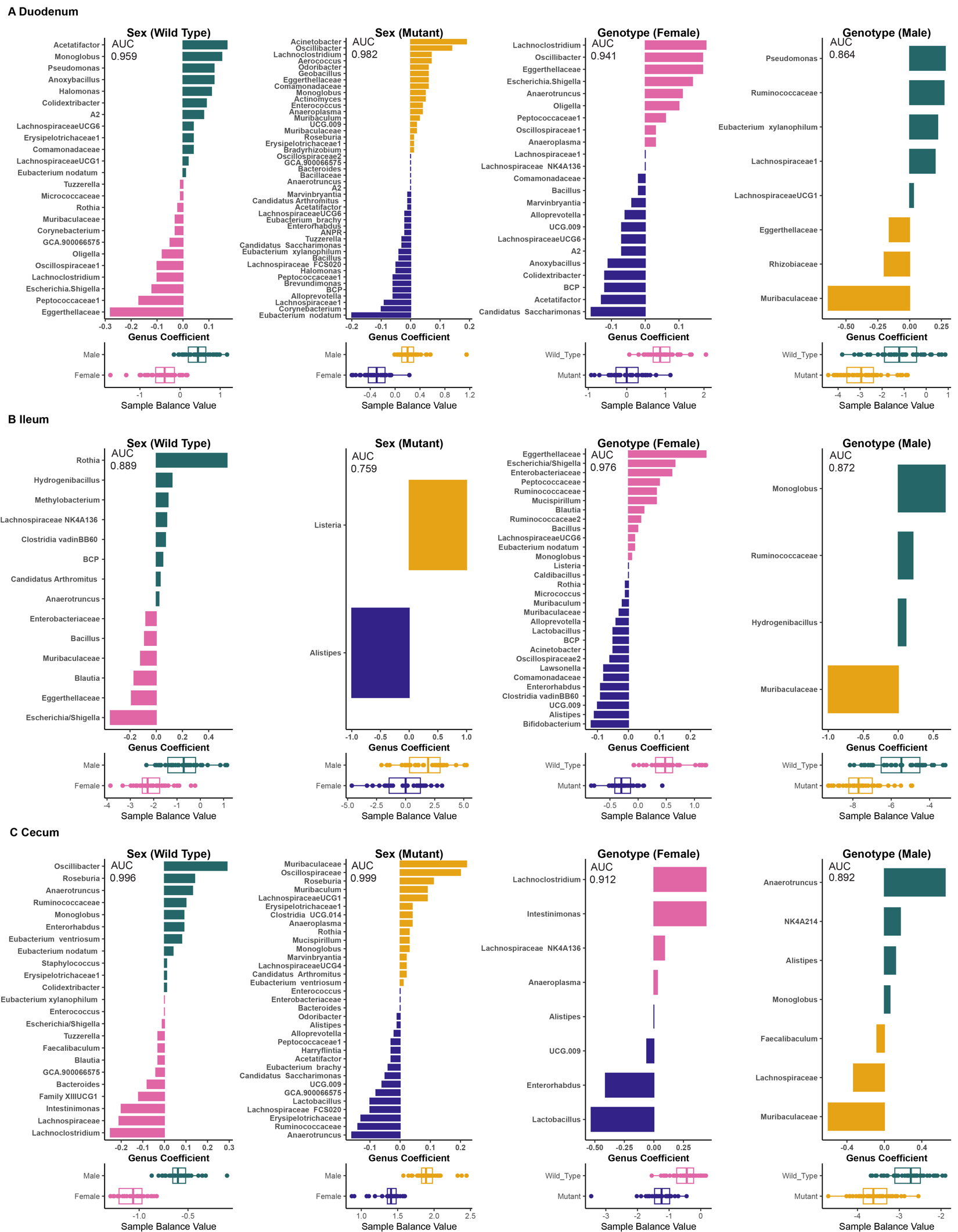
**

**Figure S6 | Strength and complexity of genus-level balance models.** Sex differences in wild-type and mutant mice and *hpg* genotype differences in female and male mice in the **A** duodenum, **B** ileum, and **C** cecum. AUC (area under the curve) indicates the model strength (AUC of 1 indicates a model with perfect predictive value). Abbreviations: ANPR genus: *Allorhizobium /Neorhizobium/Pararhizobium/Rhizobium* genus group. BCP genus: *Burkholderia/Caballeronia/Paraburkholderi* genus group.

**
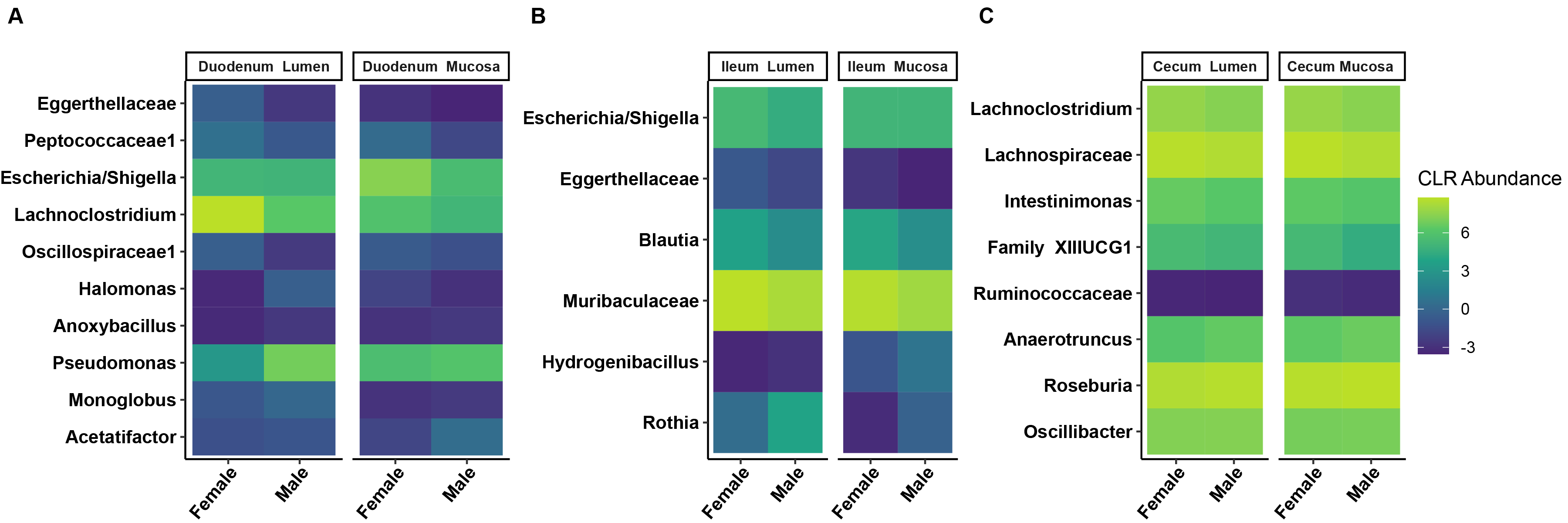
**

**Figure S7| Relative abundance of genera in *hpg* wild-type balance differs by sample type.** Heatmaps comparing genera abundances between wild-type sexes separating lumen and mucosa for the **A** duodenum, **B** ileum, and **C** cecum.
